# Silver and gold nanoparticles as a novel approach to fight Sarcoptic mange in rabbits

**DOI:** 10.1038/s41598-024-60736-w

**Published:** 2024-05-09

**Authors:** Eman I. Hassanen, Eman A. Morsy, Mai Abuowarda, Marwa A. Ibrahim, Mohamed Shaalan

**Affiliations:** 1 Department of Pathology, Faculty of Veterinary Medicine, Cairo University, P.O. Box 12211, Giza, Egypt; 2 Department of Poultry Disease, Faculty of Veterinary Medicine, Cairo University, P.O. Box 12211, Giza, Egypt; 3 Department of Parasitology, Faculty of Veterinary Medicine, Cairo University, P.O. Box 12211, Giza, Egypt; 4 Department of Biochemistry and Molecular Biology, Faculty of Veterinary Medicine, Cairo University, P.O. Box 12211, Giza, Egypt; 5 Polymer Institute, Slovak Academy of Science, Bratislava, Slovakia

**Keywords:** Biomarkers, Gene expression, Histopathology, Nanoparticles, *Sarcoptes scabiei*, Biotechnology, Drug discovery, Molecular biology, Diseases, Pathogenesis

## Abstract

Various kinds of pets have been known to contract the ectoparasite *Sarcoptes scabiei*. Current acaricides are becoming less effective because of the resistance developed by the mite besides their adverse effects on the general activity and reproductive performance of domestic pets. For this reason, the present study aims to discover a novel and safe approach using silver and gold nanoparticles to fight Sarcoptic mange in rabbits as well as to explain their mechanism of action. 15 pet rabbits with clinical signs of Sarcoptic mange that were confirmed by the microscopic examination were used in our study. All rabbits used in this study were assessed positive for the presence of different developing stages of *S. scabiei*. Three groups of rabbits (n = 5) were used as follows: group (1) didn’t receive any treatment, and group (2 and 3) was treated with either AgNPs or GNPs, respectively. Both nanoparticles were applied daily on the affected skin areas via a dressing and injected subcutaneously once a week for 2 weeks at a dose of 0.5 mg/kg bwt. Our results revealed that all rabbits were severely infested and took a mean score = 3. The skin lesions in rabbits that didn’t receive any treatments progressed extensively and took a mean score = of 4. On the other hand, all nanoparticle-treated groups displayed marked improvement in the skin lesion and took an average score of 0–1. All NPs treated groups showed remarkable improvement in the microscopic pictures along with mild iNOS, TNF-α, and Cox-2 expression. Both nanoparticles could downregulate the m-RNA levels of IL-6 and IFγ and upregulate IL-10 and TGF-1β genes to promote skin healing. Dressing rabbits with both NPs didn’t affect either liver and kidney biomarkers or serum Ig levels indicating their safety. Our residual analysis detected AgNPs in the liver of rabbits but did not detect any residues of GNPs in such organs. We recommend using GNPs as an alternative acaricide to fight rabbit mange.

## Introduction

One of the most prevalent clinical entities among rabbits and other fur-bearing animals is skin disorders^1^. The mite *Sarcoptes scabiei* is the primary cause of the highly transmissible, year-round, pruritic skin disorder known as sarcoptic mange in rabbits. *Sarcoptes scabiei* is a burrowing mite that infests the epidermis of mammals, including humans, all over the world^2,3^. In Egypt, mange infestation in rabbits has become a prevalent and fundamental problem^4^. It is a widespread rabbit disease that is horizontally transmitted from one rabbit to another. The infection produces acute itching, trembling, and scabby sores in different body areas including the ear, nose, mouth and legs, besides decreased growth^5^. The common therapy of sarcoptic mange is ivermectin, nevertheless, it has performance-related adverse effects including, testicular germ cell necrosis and azoospermia in male rabbits. Meanwhile, it causes diffuse hemorrhagic metritis and ovarian degeneration in female rabbits^6^. So, it is important to find an alternative way to control the sarcoptic mange in rabbits without affecting their performance.

Nowadays, nanoparticles, especially metal oxide and metal ions exhibit direct antimicrobial activity against a wide variety of pathogens including multidrug-resistant bacteria, fungi, and parasites^7,8^. In addition, they have a role in immunomodulation and enhancing the immunity of animals when added to their feed^9,10^. Different forms of nanoparticles were shown to be effective against internal protozoal parasites such as *Trypanosoma* spp. and *Leishmania* spp.^11,12^. Silver nanoparticles exert their antiparasitic action on insects by inhibiting their detoxifying and antioxidant enzyme system^13,14^. Moreover, silver nanoparticles disrupt the gene expression of the insect while gold nanoparticles interfere with the insect’s fertility and development by inhibiting trypsin^15^. Silver nanoparticles could act on various stages of the parasite’s life cycle as Pimentel-Acosta et al.^16^ reported their efficacy on eggs and adults of monogenean species in fish. Applications of nanoparticles as novel pesticides for external parasites such as ticks and mosquitoes have been studied recently, yet there is not enough information about their mechanism of action on mites^14^. Therefore, the present study aims to discover novel antiparasitic nanoparticles to fight sarcoptic mange in rabbits without any side effects along with a comprehensive insight to study their mechanism of action, especially the molecular pathway.

## Materials and methods

### Synthesis and characterization of nanoparticles

We previously synthesized AgNPs suspension (17 ± 5 nm) by a co-precipitation method via the chemical reduction of silver nitrate (AgNO_3_) with trisodium citrate dehydrate and sodium borohydride (99%, Aldrich, US) with continuous stirring (99.99%, Aldrich, US), and GNPs by a chemical reduction of Gold (III) chloride hydrate (99.995%, Sigma-Aldrich, St. Louis, MO, USA) with addition of Tri-sodium citrate dihydrate (99%, Sigma-Aldrich, St. Louis, MO, USA) as a reducing agent during boiling^17,18^. Additionally, both nanoparticles were previously identified by several methods including a Nanodrop UV–Visible spectrophotometer, Transmission Electron Microscope (TEM) operating at an accelerating voltage of 200 kV (Tecnai G2, FEI, Netherlands), and zeta sizer (Malvern, ZS Nano, UK).

### Animals and treatment

All procedures were designed following the ARRIVE guidelines (PLoS Bio 8(6), e1000412, 2010) and conducted in accordance with the eighth edition (2011) of the rules for the care and use of laboratory animals. They were all authorized by the Institutional Animal Care and Use Committee at Cairo University IACUC (Vet CU 8032022428), Cairo, Egypt.

The current study was performed on 15 pet rabbits weighing 4–5 kg obtained from local farms (Sharkia, Egypt), where rabbits exhibited severe clinical signs of acute pruritis, alopecia, and erythema with the presence of white dried crusted scab on the ear pinna, face, nose, and legs. All rabbits were subjected to the microscopic examination of deep skin scrapings to ensure their infestation with Sarcoptic mange. It was previously mentioned that these rabbits did not respond to any treatment of antibiotics or acaricides either topically applied or injected. During the investigation period, all rabbits stayed under their common housing circumstances, individually cage housed, and were supplied by well-balanced commercial ration and normal potable water ad-libitum.

All rabbits were randomly divided into three groups of five rabbits each as follow: group (1) didn’t receive any treatment and was kept as a control positive group, and group (2 and 3) was treated with AgNPs and GNPs, respectively. Both nanoparticles were applied daily on the affected skin areas via dressing as well as injected subcutaneously once a week for 2 weeks at a dose of 0.5 mg/kg bwt. The dose of nanoparticles was selected according to previous studies which confirmed that the dosage level of 0.5 mg/kg bwt from both AgNPs and GNPs didn’t induce any toxicological evidence in rabbits^19,20^. Since we didn't create an experimental infection and the study was done on rabbits infected naturally with sarcoptic mange, we did not include a control negative non-infected group in this investigation to minimize the number of rabbits following the rules of animal welfare.

All rabbits were observed daily to determine either the progression or improvement in the skin lesions of different treatment groups. 5-pointed scales were utilized to assess the severity of skin lesions in diverse groups according to the method described by Casais et al.^21^ and Singh et al.^22^. The invasion of skin lesions was indexed as follows: 0, normal skin; 1, erythema and dryness of skin (mild lesion ≤ 7 cm^2^); 2, erosion or ulceration with severe exudation (moderate lesion 7–15 cm^2^); 3, severe alopecia with crust formation (severe lesion 15–31 cm^2^); 4, obvious crust and hyper keratinization spread to different skin parts (extensive severe lesion > 31 cm^2^).

### Sampling

Skin scrapes were taken aseptically from each rabbit twice a week all over the experimental period using a blunt scalpel blade dipped in liquid paraffin from approximately 2.5 cm^2^ skin lesions in three distinct affected locations. To break down the skin debris, the scrapes were treated with 10% KOH and heated. After centrifugation, the sediment was examined under light microscopy for the presence and identification of mites^23^.

Blood samples were collected from the ear vein of each rabbit after 21 days post-treatment. To obtain clear serum samples, centrifugation was done at 4500 rpm for 5 min, then preserved at − 20 °C until needed for the examination of biochemical parameters. After 21 days, all rabbits were anesthetized by using intramuscular injections of ketamine (30 mg/kg) and xylazine (5 mg/kg) and then euthanized by exsanguination to get samples from the skin, muscles, and liver^24^. For histology and immunohistochemistry, a small portion of the skin samples were fixed in 10% neutral buffered formalin, while the remaining samples were preserved at − 80 °C for Rt-PCR and residual analysis.

### Parasitological examination

Skin scrapings were spread on microscope slides with petroleum jelly and examined under light microscopy (40×). If adult mites were detected, the following morphological characteristics with taxonomic value were identified utilizing the techniques explained by Flynn^25^ and Suckow et al.^26^.

### DNA extraction from collected mites

Sarcoptes mites were isolated from rabbits and were kept in storage at − 80 °C. Following the manufacturer's instructions, we extracted DNA for PCR using the techniques outlined by Walton et al.^27^ and the DNeasy Tissue Kit (Qiagen, Düsseldorf, Germany). The forward primer F (5′-CTAGGGTCTTTTTGTTCTTGG-3′) and the reverse primer R (5′-GTAAGTATACGTTGTTATAAC-3′) were used in a PCR reaction to analyze the target areas of the small subunit of 16S rRNA of zoonotic Sarcoptidae^28^. These primers are intended to produce 371 bp amplicons.

### Analysis using phylogenetics and DNA sequencing

A QIAquick purification kit (Qiagen, Germany) was used to purify the positive PCR results of the mite in accordance with the manufacturer's instructions. The Big Dye Terminator V3.1 kit from Applied Biosystems was used to perform one-direction Sanger sequencing on the purified products in the ABI 3500 Genetic Analyzer (Applied Biosystems, USA). Then, the BLAST server on the NCBI website was used to analyze the similarity of the nucleotide sequences of the PCR results to those available in the GenBank. The obtained sequences were compared to sequences isolated from rabbits and other animals around the world as well as sequences documented in Egypt. The Clustal W, BioEdit software (ver. 7.0.9) was used for the analysis^29^. The phylogenetic tree was created by using the Maximum Likelihood approach and the Tamura–Nei model using Mega 6.06 software, and bootstrap analysis with 1000 replicates was obtained. With the use of the DNASTAR program (Laser gene, version 8.0), a similarity matrix was conducted. The Meg-Align project of DNSTAR software was used to analyze the genetic distance values of species variants^30^.

### Accession numbers for nucleotide sequences

A partial 16S rRNA *S. scabiei* gene sequence from adult rabbit mites was uploaded to GenBank, where the following accession number (ON241779) and sequence length (371 bp).

### Biochemical parameters

Using standard kits marketed by (SPECTRUM-Germany) in accordance with the manufacturer's guidelines, the following enzymes were measured in the collected serum samples: alanine aminotransferase (ALT), aspartate aminotransaminase (AST), total proteins (TP), albumin, urea, and creatinine. The immunoglobulin levels were measured via ELISA technique using rabbit polyclonal antibodies against IgG and IgM as described in the manufacturer kit (Biocheck Inc., Foster City, CA, USA). Utilizing inductively coupled plasma atomic emission spectrometry (ICP-AES) at wavelengths of 213.856 nm, zinc levels were determined.

### Histopathological examination

Formalin-fixed skin tissue samples were traditionally processed using an ascending grade of ethanol for water removal, purified by Xylene, soaked in paraffin wax, and cut into 4.5 μm sections which were stained by H&E to inspect them under a light Olympus microscope for any histological changes^31^.

A quantitative grading method was done to measure the extent of distribution of the observed microscopic skin lesion according to the protocol explained by Espinosa et al.^32^. The scoring was blindly performed in five randomly selected microscopic fields/slides in a total of three slides/groups. The epidermal changes were evaluated by counting the average number of mites and/or burrows, epidermal cell layers, vacuolated cells, necrotic cells, and exocytosis foci in diverse groups. Additionally, the counts of dermal inflammatory cell infiltration (neutrophils, eosinophils, mast cells, lymphocytes, and plasma cells) were also estimated at high-powered fields (400×).

### Immunohistochemical staining

Avidin–biotin-peroxidase detecting technique (ABC) was applied to localize inducible nitric oxide synthetase (iNOS), cyclooxygenase-2 (Cox-2), and tumor necrosis factor-alpha (TNF-α) in the skin tissue sections. Dewaxed, dried skin tissue slices were first incubated with various primary antibodies (Abcam Ltd., USA), and then the ABC reagents (Vector Laboratories' Vectastain ABC-HRP Kit) were applied. Slides were then dyed using DAB-chromogen substrate (Sigma), followed by examination under a light Olympus microscope. The immunopositivity area was determined in relation to the total area of target cells by utilizing Image J software.

### Quantitative RT-PCR analysis for IL-6, IL-10, TGF-1β, and IFγ

The RNeasy Mini Kit (Qiagen Cat No./ID: 74104) was used to extract the total RNA from the samples. We examined the concentration and purity of the isolated RNA using NanoDrop. The *SuperScript Reverse Transcriptase* (Thermo Scientific) was used to synthesize the first strand c-DNA following the manufacturer’s protocol. The SYBR™ Green PCR Master Mix (Thermo Scientific Cat number: 4309155) was used to perform the qRT-PCR in the ABI Prism StepOnePlus Real-Time PCR System (Applied Biosystems) following the manufacturer’s protocol. The primer sequences of the target genes were listed in Table 1. The relative m-RNA expression levels were normalized to the GAPDH which is used as an internal control.

**Table 1:** The primer sets of the studied genes.

Gene	Forward primer	Reverse primer	Product	Accession no
TGF-1	GATGAATCCTCTGGTGCGTCTC	GCTGTGTGGCTAAGGTTCCA	135	XM_017341072.1
IFγ	GACCCTCCTGTCACTTCGAC	TGTACGATTGTTCAGGCCCA	181	NM_001081991.1
IL6	GCCAAGTTCAGGAGTGACGA	AGAGCCCATGAAATTCCGCA	291	NM_001082064.2
IL10	AAAAGCCCCAGGATGGCAAC	CACCCCATGGGAACAGCTTA	247	NM_001082045.1
GAPDH	CGAGCTGAACGGGAAACTCA	CCCAGCATCGAAGGTAGAGG	229	NM_001082253.1

*TGF-1* transforming growth factor 1, *IFγ* interferon gamma, *IL6* interleukin-6, *IL10* interleukine-10, *GAPDH* glyceraldehyde-3-phosphate dehydrogenase.

### Residual analysis of silver and gold in the most edible parts

Residues of both silver and gold were measured by inductively coupled plasma mass spectroscopy (ICP-MS) in the most edible parts including muscle and liver that collected from rabbits in diverse groups following the keys described by Hassanen et al.^33^.

### Statistical analysis

All the parametric values were expressed as means ± standard error of the mean (SEM). The statistical package program (SPSS version 25) was used to analyze the recorded results using one-way analysis of variance (ANOVA) and post hoc Duncan's test; *P* values ≤ 0.05 are considered statistically significant. The Mann–Whitney *U* test was used after the Kruskal Wallis H test to analyze nonparametric values for the skin lesion score.

### Compliance with ethics requirements

All experimental protocols were approved by a named institutional and/or licensing committee (Institutional Animal Care and Use Committee at Cairo University IACUC (Vet CU 8032022428), Cairo, Egypt).

## Results

### Nanoparticle characterization

**Figure 1:**
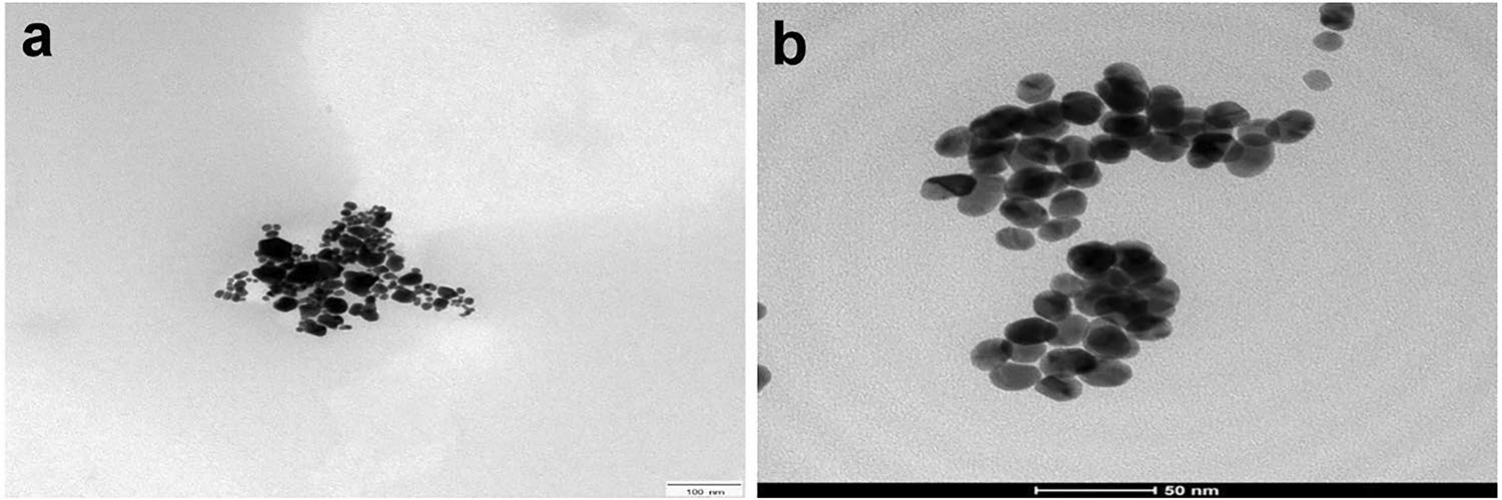
HR-TEM image showing spherical shaped particles of AgNPs (scale bar = 100 nm) (**a**), and GNPs (scale bar = 50 nm) (**b**).

The findings of both nanoparticles’ characterization were illustrated in Fig. 1 and summarized in Table 2.

**Table 2:** Characterization of the prepared nanoparticles.

	AgNPs	GNPs
UV–Visible spectrophotometer	Maximum absorbance 0.528 at a wavelength of 400 nm	Maximum absorbance 0.97 at a wavelength of 530 nm
Particle size	23.4 nm	17.5 nm
Zeta potential	− 22 mV	− 30.4 mV
HR-TEM image	Spherical shaped particles	Spherical shaped particles

### Clinical signs and skin lesion scoring

**Figure 2:**
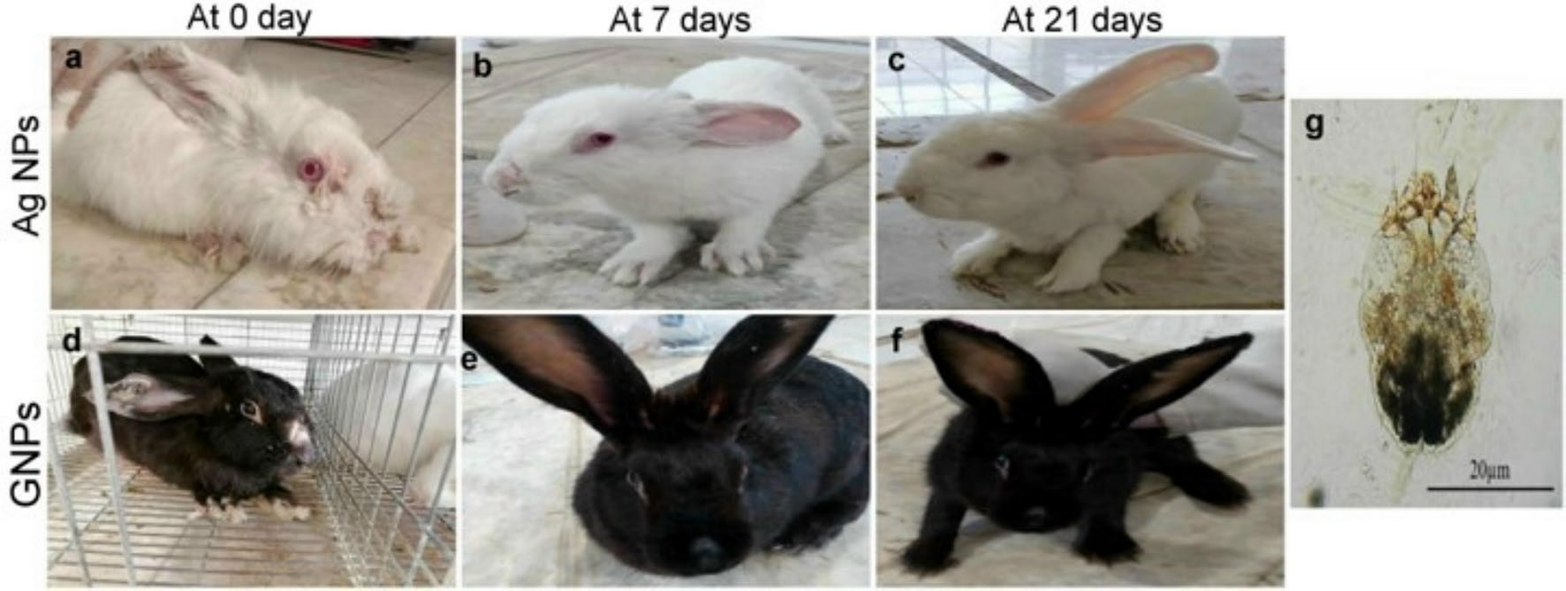
(**a**–**f**) Photographs of the gross skin lesions of Sarcoptic mange in rabbits represented both nanoparticles treatment groups. (**a**–**c**) group treated with Ag NPs and (**d**–**f**) group treated with GNPs. (**a**,**d**) Rabbits with severe skin lesions of Sarcoptic mange on the nose, toes, and external edge of the ear at 0 day. (**b**,**e**) The same rabbits at 7 days post-treatment showed falling of all overgrowth hyper-keratinized tissues and healing of the skin lesions. (**c**,**f**) Rabbits after 21 days post-therapy showed complete recovery. (**g**) Microphotograph of a *Sarcoptes scabiei* mite detected from the skin scrapes of a diseased rabbit.

Anorexia, severe itching, erythema, alopecia, hyper keratinization, and white dried crusted scabs on different skin areas including the ear pinna, around the eye, nose and toes were among the clinical symptoms seen in rabbits enrolled in this study. All rabbits were severely infested (score = 3) at 0 days and progressed extensively (score = 4) in the untreated groups throughout the experiment. On the other hand, all nanoparticle treatment groups displayed marked improvement in the skin lesion. We noticed a quick decrease in itching and an increase in the rabbits' vitality and appetite with the falling of all overgrowth hyper-keratinized tissues after 7 days from the treatments. Meanwhile, a complete resolution of the skin lesions along with a significant improvement in the general activity of rabbits was seen after 21 days of the treatments (Fig. 2a–f). The best improvement was recorded in the GNPs receiving group which takes a score = of 0 followed by the AgNPs receiving group which takes a score = of 1.

### Parasitological examination

All rabbits used in this study were assessed positive for the presence of different developing stages of *S. scabiei* (Fig. 2g), while mites were found to be absent in all nanoparticles-treated rabbits after 7 days of the treatment.

### Molecular findings and sequencing analysis

**Figure 3:**
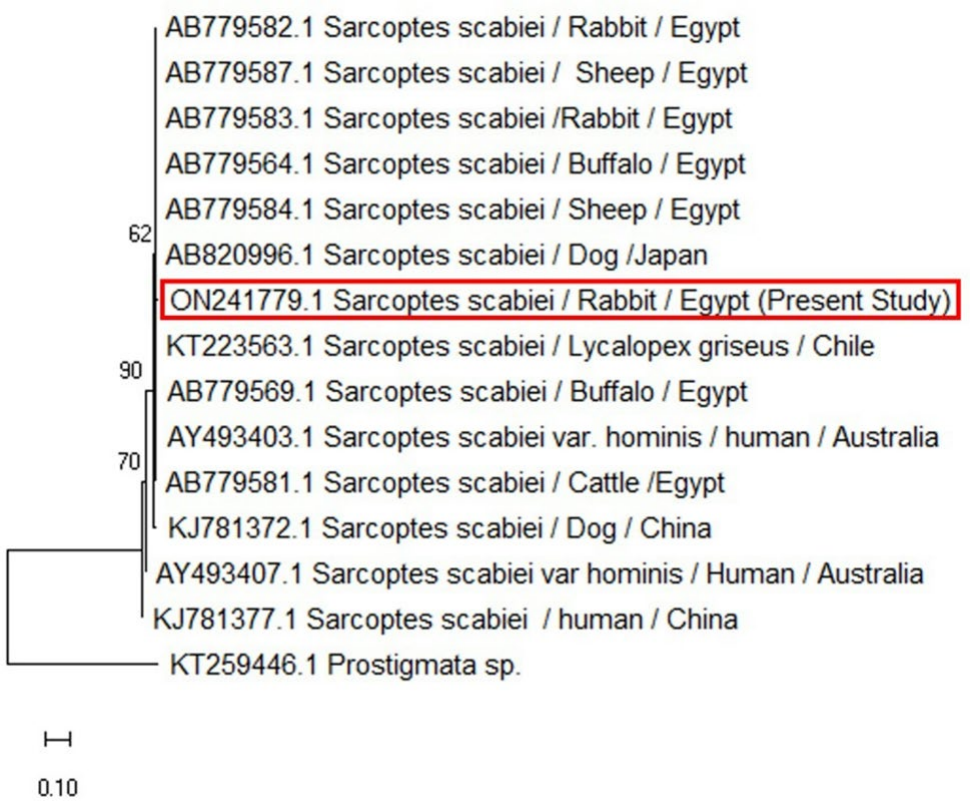
Phylogenetic relationships based on the small subunit of 16S ribosomal RNA (16S rRNA) sequences of *Sarcoptes scabiei*. The tree was constructed and analyzed using the Maximum Likelihood method and Tamura–Nei model and numbers above internal nodes show the percentages of 1000 bootstrap replicates that supported the branch. Prostigmata sp. (KT259446) was considered as an outgroup.

**Figure 4:**
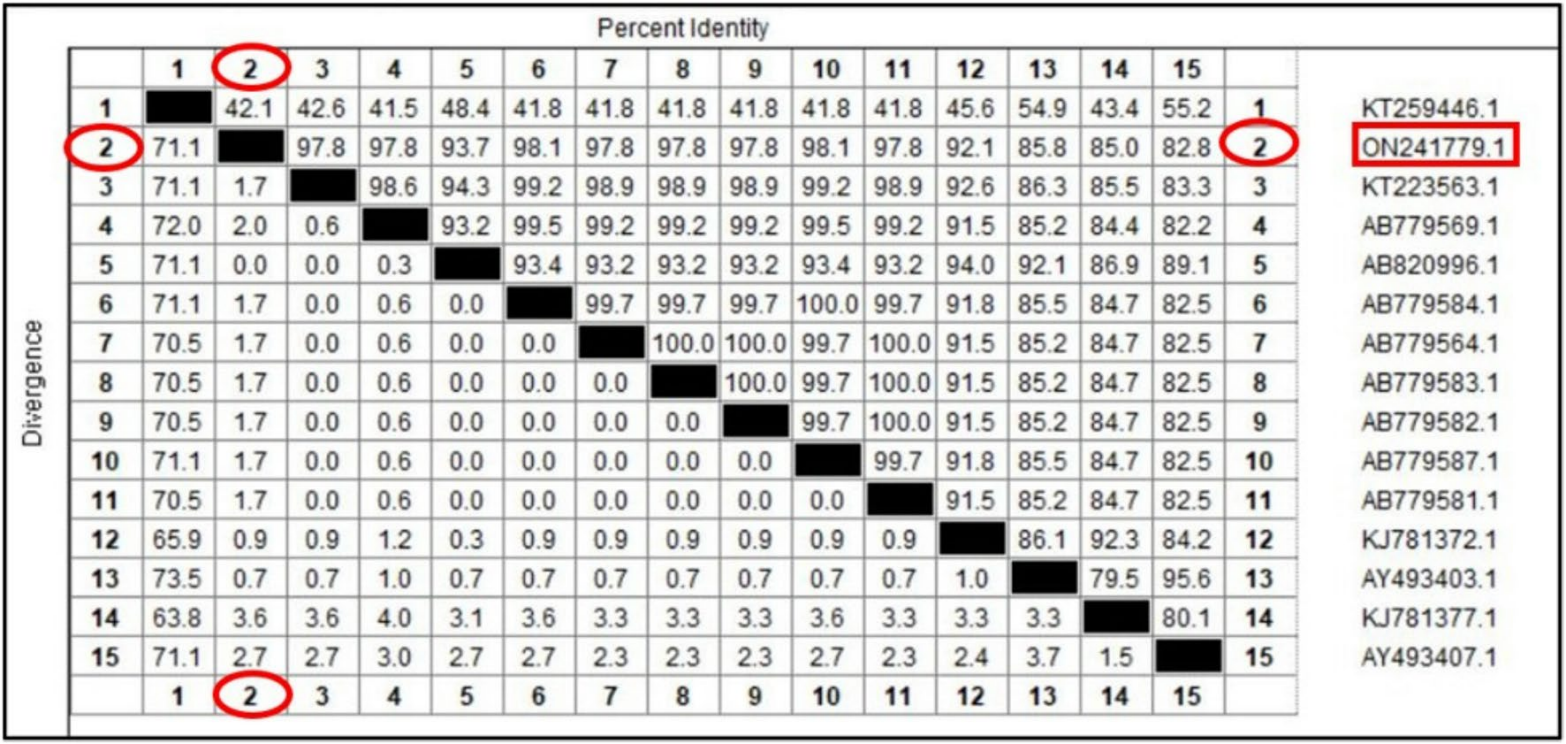
Similarity (percent identity) and genetic divergence of small subunit 16S ribosomal RNA (16S rRNA) sequences of *Sarcoptes scabiei* isolated from Rabbits in Egypt (representing number, 2) compared with the most similar reference sequences (GenBank).

The predicted amplicon molecular weight of the amplicon (371 bp) was present in all adult mites. Sequence analysis of the isolated positive samples was identical and showed a 99.72% identity to isolates of *S. scabiei* that were deposited into the GenBank database. The genetic distance between the obtained sequence and the other 15 aligned sequences of *S. scabiei* that are available in the NCBI GenBank database were analyzed based on inter- and intra-species analysis (Fig. 3). The genetic identity of *S. scabiei* revealed high sequence homology (98.1% similarity) with *S. scabiei* isolated from sheep in Egypt (Accession numbers: AB779584.1, AB779587), as well as 97.8% similarity with *S. scabiei* isolated from rabbits in Egypt (Accession number: AB779583) (Fig. 4). The similarity of isolated *S. scabiei* from rabbits with *S. scabiei* var hominis isolated from human in Australia (AY493403) was 85.8%. Based on interspecies study using genetic distance values, it was revealed that the *S. scabiei* genospecies isolated from a dog in Japan (Accession number AB820996) had zero levels of genetic divergence (GD). Furthermore, the *S. scabiei* was genetically distant (GD 1.7) from the *S. scabiei* from rabbits in Egypt (Accession numbers AB779583).

### Biochemical parameters

Serum levels of all biomarkers were within the normal acceptable limits in all groups. Administration of both silver and gold nanoparticles didn’t influence any significant difference in ALT, AST, TP, albumin, urea, creatinine, IgG, IgM, and zinc levels in contrast to the untreated group (Table 3).

**Table 3:** Effect of AgNPs and GNPs on some biochemical parameters in the serum of rabbits.

	Normal levels in rabbits	Untreated group	AgNPs group	GNPs group
ALT	45–80 IU/L	58.3 ± 6.9	71.7 ± 2.2	64.3 ± 15.6
AST	35–130 IU/L	65.3 ± 7.8	65.6 ± 5.2	59.7 ± 11.7
TP	5.4–7.5 g/dL	6.6 ± 0.8	8.2 ± 0.1*	6.4 ± 0.1
Urea	20–45 mg/dL	50.3 ± 2.3	22.3 ± 2.3*	20.7 ± 13.2*
Creatinine	0.5–2.5 mg/dL	1.1 ± 0.1	0.7 ± 0.1*	0.8 ± 0.0*
Zn	70–180 ug/100 mL	163.3 ± 2.3	140 ± 23.1	148.7 ± 10.1
IgG	5–10 mg/mL	8.5 ± 1.8	9.9 ± 0.9	7.4 ± 1.1
IgM	0–0.5 mg/mL	0.2 ± 0	0.1 ± 0	0.3 ± 0.1

Data were presented as mean ± SEM (n = 5), all values were compared with the normal acceptable limits in rabbits that were mentioned in the manufacturer kits.

*ALT* alanine aminotransferase, *AST* aspartate aminotransferase, *TP* total protein, *Zn* Zinc, *IgG* immunoglobulin G, *IgM* immunoglobulin M.

*Means a significant difference versus the untreated group at *P* ≤ 0.05.

### Histopathological examination

**Figure 5:**
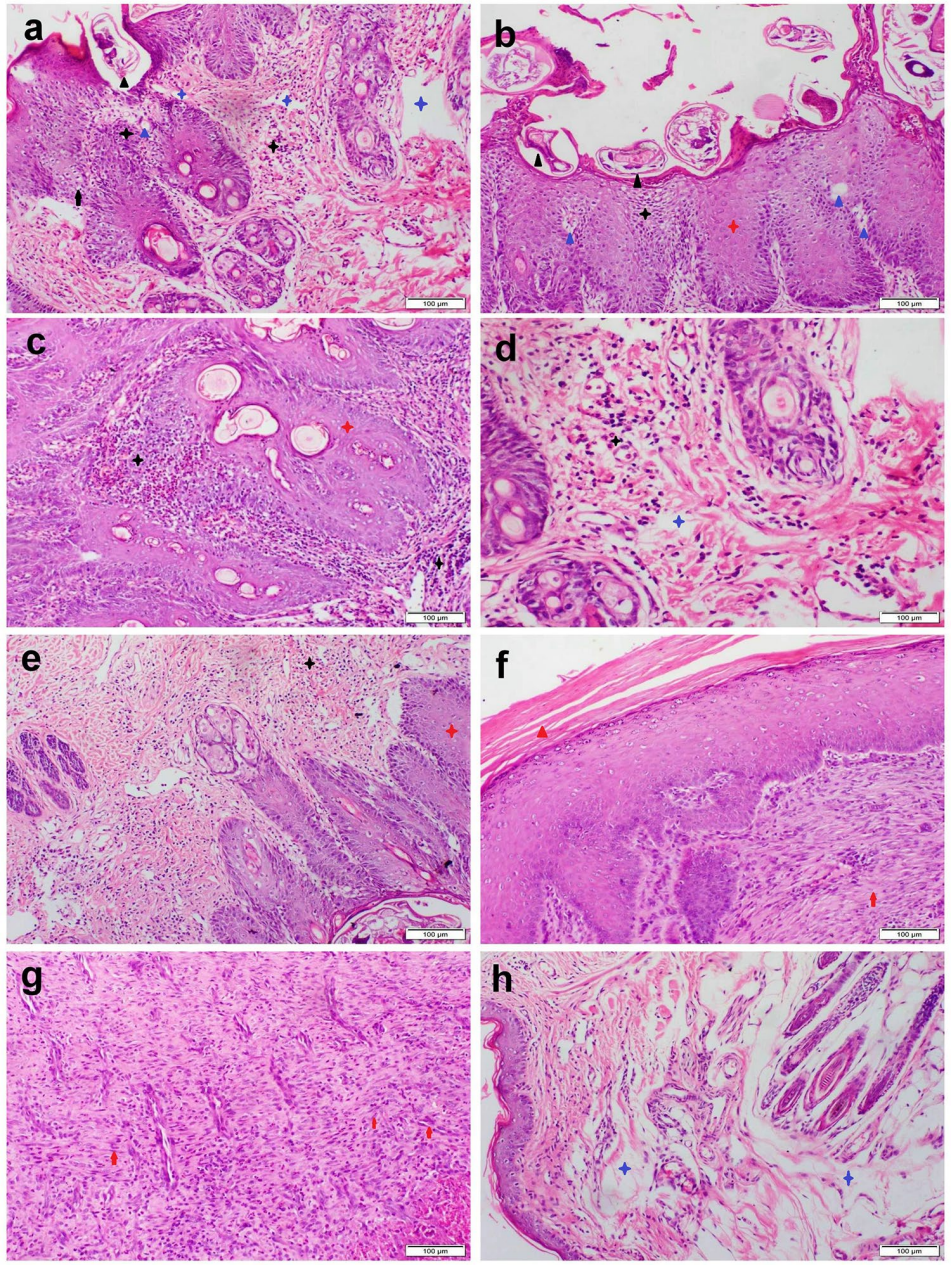
Photomicrograph of skin sections stained with H&E that represented diverse groups. (**a**–**d**) untreated infected rabbit demonstrated epidermal hyperplasia (red stars), numerous mites/burrows (black triangles), spongiosis (blue triangles), vacuolar degeneration (black arrows), inflammatory cells infiltration (Black stars), and dermal edema (blue stars). (**e**–**g**) AgNPs treated group demonstrated mild hyperkeratosis (red triangle), epidermal hyperplasia (red stars), moderate dermal inflammatory cells infiltration (Black stars), and dermal organized tissue formation (red arrows). (**h**) GNPs treated group demonstrated normal epidermal layer and moderate dermal edema (blue stars).

Skin sections obtained from the untreated infected rabbits showed extensive histopathological alterations in the epidermal and dermal layers. There were severe epidermal hyperplasia, acanthosis, parakeratosis, and hyperkeratosis along with extensive spongiosis (Fig. 5a). An enormous number of mites and/or burrows were seen in most sections (Fig. 5b). Dermis showed extensive congestion and inflammatory cells infiltration (Fig. 5c). The main inflammatory cells observed are eosinophils, plasma cells and lymphocytes mixed with other cells including neutrophils and macrophages (Fig. 5d). Most of the hair follicles showed folliculitis and converted into small sacs filled with inflammatory cells. There were moderate improvements in the epidermal and dermal lesions of both nanoparticle-treated groups. AgNPs treatment groups displayed moderate to a marked improvement in skin lesions in all treated cases. Some cases showed mild epidermal hyperplasia and hyperkeratosis along with moderate mixed inflammatory cells infiltration within the dermis (Fig. 5e). Other cases showed normal epidermal histology with the proliferation of fibroblast and angioblast in the dermal layer forming organized tissue (Fig. 5f,g). Otherwise, the GNPs receiving group showed the normal histological structure of both epidermis and dermis except for mild dermal edema and lymphocytic exocytosis (Fig. 5h).

The outcomes of the quantitative grading were outlined in Table 4. In the epidermis, there was a marked decline in the number of mites/burrows, epidermal layers, exocytic cysts, spongiotic and necrotic cells in both NPs treated groups in contrast to the untreated group but the best improvement was recorded in the GNPs group in comparison with AgNPs group. Regarding the dermal lesion grading, a notable decline in the total number of heterophiles, eosinophils, plasma cells and lymphocytes was recorded in both NPs treated groups along with a mild increase in mast cell counts in contrast to the untreated group.

**Table 4:** The quantitative scoring of the skin lesion in different treatment groups.

	Untreated group	AgNPs group	GNPs group
Epidermal lesion scoring			
Mites/burrows/field	8 ± 2.1^a^	2.3 ± 0.5^b^	0
Epidermal layer	43 ± 6.3^a^	13 ± 1.4^b^	5 ± 1.1^c^
Spongiotic cells/mm^2^	15 × 10^4^ ± 8602^a^	8 × 10^2^ ± 58^b^	0
Necrotic cells/mm^2^	9 × 10^4^ ± 6615^a^	0	0
Exocytotic cyst/field	13 ± 1.5^a^	3 ± 0.2^b^	0
Dermal lesion scoring			
Heterophiles/mm^2^	1.5 × 10^4^ ± 995^a^	0	0
Eosinophils/mm^2^	13 × 10^4^ ± 5010^a^	100 ± 2.5^b^	0
Mast cells/mm^2^	8 × 10^3^ ± 520^a^	100 ± 1.6^b^	100 ± 2.3^b^
Plasma cells/mm^2^	25 ± 3.3^a^	0	0
Lymphocytes/mm^2^	15 × 10^4^ ± 7700^a^	10 × 10^3^ ± 639^b^	100 ± 5.7^c^

Values are presented as mean ± SEM (n = 15). Values denoted with different superscript letters (a, b, c) in the same row are statistically significant at *P* ≤ 0.05.

### Immunohistochemical staining

**Figure 6:**
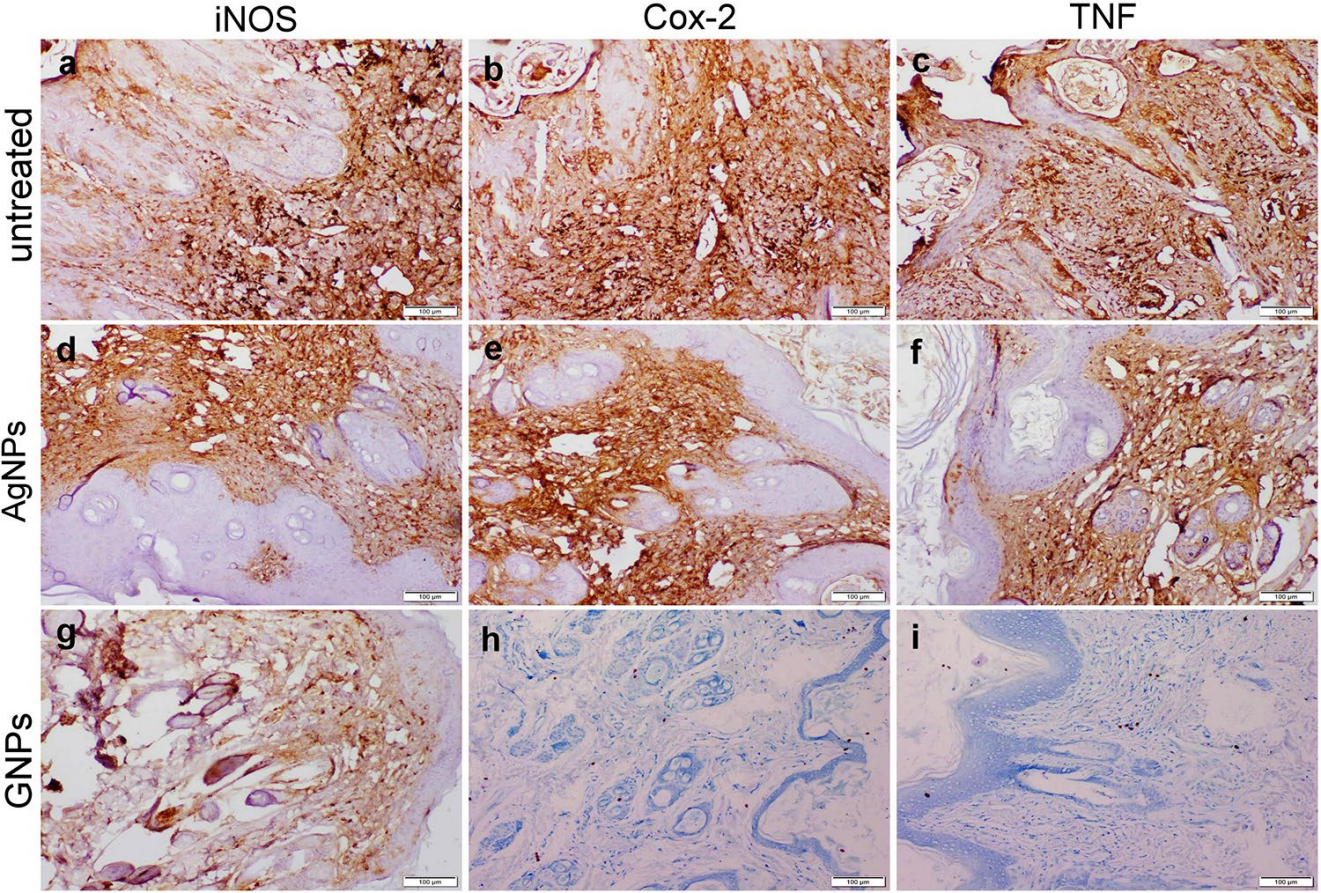
Photomicrograph of skin sections represented iNOS, Cox-2, and TNF-α immunoexpression in diverse groups. (**a**–**c**) untreated infected rabbit displayed strong positive immunoexpression of all studied markers. (**d**–**f**) AgNPs treated group exerted moderate immunopositivity of all mentioned markers. (**g**–**i**) GNPs treated group showed weak iNOS immunoexpression along with negative Cox-2 and TNF-α immunoexpression.

Skin sections of rabbits in the untreated group showed strong iNOS, Cox-2, and TNF-α immunopositivity in all skin sections. On the other hand, both nanoparticles treatment groups displayed negative to mild positive reactions to the previously mentioned immune markers (Fig. 6). Image analysis recorded the highest immunostaining area of the studied immune markers in the skin of the untreated rabbits, while the mean percentage areas of positive immunoexpression were markedly declined in both nanoparticles treated rabbits. The lowest positive immunostaining area was noticed in the GNPs-treated group (Table 5).

**Table 5:** Mean % area of iNOS, Cox-2 and TNF-α in the skin tissue of diverse groups.

	Untreated group	AgNPs group	GNPs group
INOS	35 ± 3.5^a^	20 ± 0.9^b^	9 ± 2.3^c^
Cox-2	40 ± 5.7^a^	15 ± 1.5^b^	0
TNF-α	25 ± 2.4^a^	9 ± 1.2^b^	0

Values are presented as mean ± SEM (n = 5). Values denoted with different superscript letters (a, b, c) in the same row are statistically significant at *P* ≤ 0.05.

### Quantitative RT-PCR analysis for IL-6, IL-10, TGF-1β, and IFγ

**Figure 7:**
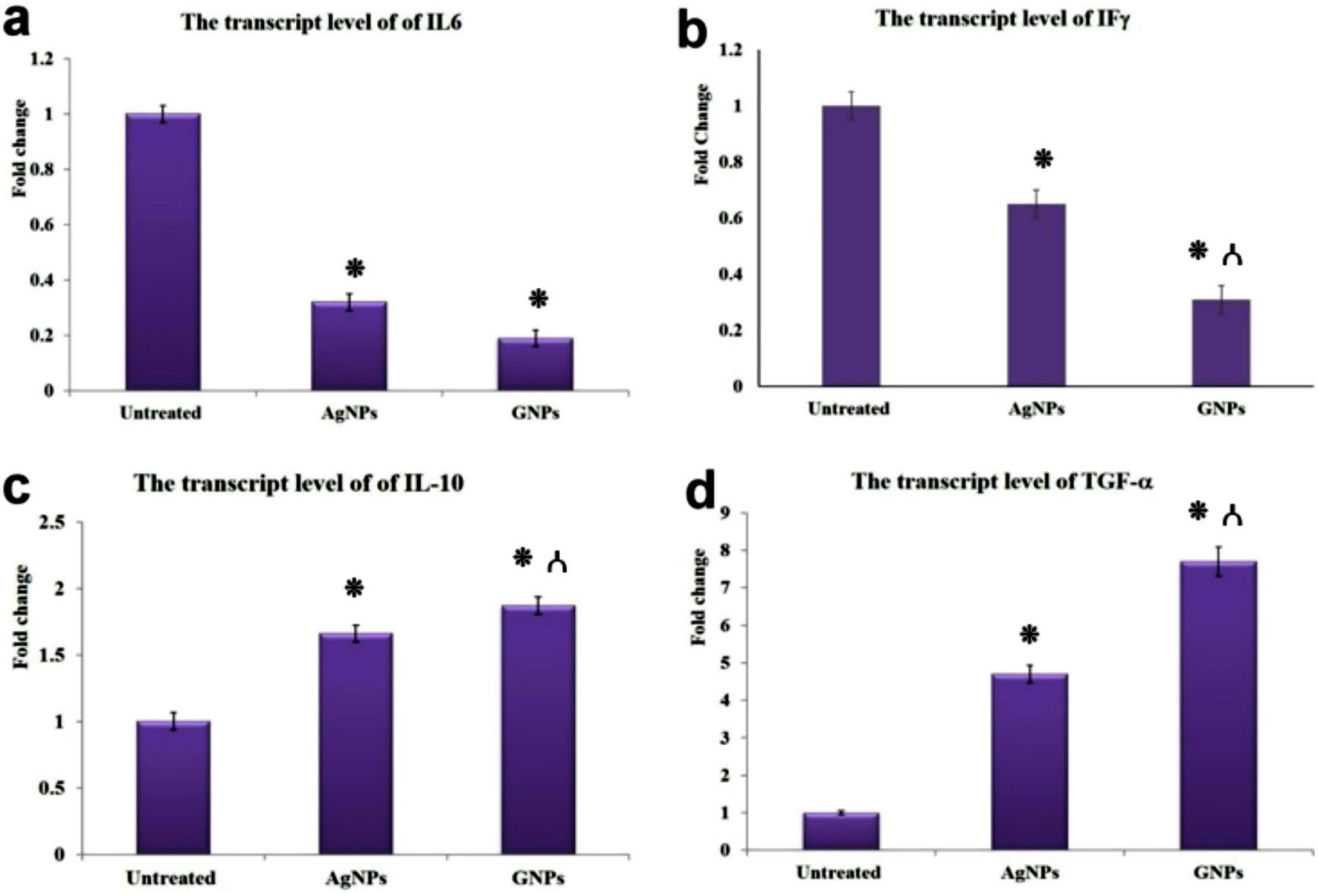
Bar charts represented the transcriptase levels of IL-6 (**a**), IFγ (**b**), IL-10 (**c**), and TGF-1 (**d**) in diverse groups. Values are presented as mean ± SEM (n = 5). (⁕) Significant difference vs control group at *P* values ≤ 0.05, () significant difference versus AgNPs group at *P* values ≤ 0.05.

There was a significant upregulation of m-RNA levels of IL-10 and TGF-1β with downregulation of IL-6 and IFγ genes in both nanoparticles treated groups in contrast to the untreated group. Meanwhile, the best results were recorded in the GNPs-treated group compared with the AgNPs-treated group (Fig. 7).

### Residual analysis of silver and gold in the most edible parts

ICP-MS revealed a significant increase in the liver content of AgNPs in the AgNPs receiving group in contrast to the untreated group. Otherwise, the muscular samples obtained from AgNPs receiving group didn’t record any increase in AgNPs. Otherwise, there were no detectable levels of GNPs in the liver and muscles obtained from GNPs receiving group (Table 6).

**Table 6:** Residual analysis of both nanoparticles in diverse groups.

	Untreated group	AgNPs group	GNPs group
Muscular content of both nanoparticles			
AgNPs (ppp)	0	0.05 ± 0.02*	0
GNPs (ppp)	0	0	0
Liver content of both nanoparticles			
AgNPs (ppp)	0	35 ± 1.5*	0
GNPs (ppp)	0	0	0

Values are presented as mean ± SEM (n = 5).

*Means a significant difference vs the untreated group at *P* ≤ 0.05.

## Discussion

Rabbits are universally used as pets, food animals, and experimental animals. Because of their fast growth and reproduction, they are an excellent supplier of low-cost but high-quality meat. *Sarcoptic scabiei* mange is an extremely contagious and highly transmissible disease^34^, that can jeopardize the health and immunity of rabbit breeders^35^. The accuracy of the standard *S. scabiei* diagnostic tests is under 50%^36^. So, Molecular characterization of the 16S rRNA gene of *S. scabiei* was necessary to diagnose and find mite species. It is an accurate, credible, and sensitive approach^37^ as revealed in the current study by amplification of specific genes of *S. scabiei*. This approach can be useful in developing future risk assessment systems that consider rabbits as potential infestation reservoirs. The genetic identity of *S. scabiei* showed a high sequence homology (98.1% similarity) with the *S. scabiei* (Accession number: AB779584.1, AB779587) isolated from a sheep in Egypt and 97.8% with *S. scabiei* isolated from rabbits in Egypt (Accession number AB779583).

Currently, there are few vigorously tested treatment options for scabies in rabbits, including the use of ivermectin, doramectin, and selamectin^38,39^. These drugs need to be administered multiple times (4–6 times 10–30 days apart) to treat affected rabbits, which is stressful for the animals^40^. Longer duration of treatment may also result in longer infection of the owner/contact animals and contamination of the environment with mites. Moreover, the development of *S. scabiei* resistance is an addition to available acaricides of greatest concern^41^. So, this is the first study to show that silver and gold nanoparticles can be used as a therapeutic agent for rabbits infected with spontaneous severe and moderate sarcoptic mange.

In the current study, the ear margins, nose, face, and legs of all rabbits had dried crusted scabs, according to a clinical assessment. Additionally, skin was erythematous, swollen, and had patchy hair loss, these lesions are suggestive to Sarcoptic mange in rabbits and in accordance with lesions recorded by Sharun et al.^1^. The histopathological results demonstrated adverse pathological changes in both dermal and epidermal layers of the skin tissues obtained from the untreated group. The lesions included a severe degree of inflammations associated with a high degree of epidermal hyperplasia and hyperkeratosis which agreed with earlier results from Abdelaziz et al.^42^.

Currently, a wide range of pathogens including parasites, are directly inhibited by nanoparticles, particularly metal oxide, and metal ions^7^. Recently, it has been reported that several metallic and metal oxide nanoparticles such as MgO, CuO, TiO_2_ and ZnO were toxic to mites^43,44^. In our study, the successful synthesis of AgNPs and GNPs was confirmed by UV–Visible spectroscopy which showed a peak of absorbance at 400 and 530 nm, respectively, which lies in the wavelength range for both nanoparticles^45,46^. In the current study, both AgNPs and GNPs gave promising results in the treatment of sarcoptic-infected rabbits. All treated rabbits were negative for the presence of mites on day 14 post-therapy. Concerning the microscopic appearance of skin tissues, both AgNPs and GNPs treatments caused a marked reduction in the pathological lesions as compared to the infected untreated group. To compare both treatments, GNPs show a higher improvement in skin histology than AgNPs. On the contrary, other studies showed that AgNPs had more potent action against parasites such as malaria^47^. Their mechanism of action differs, as GNPs inhibit trypsin and interfere with insect fertility and development, while AgNPs impair the insect's gene expression^14^. Additionally, AgNPs inhibited the insects' detoxification and antioxidant enzyme systems, which thus have an antiparasitic effect on them^11^. Several processes that NPs can go through following their entrance include phagocytosis, deposition, clearance, and translocation. They can bind to and interact with proteins.

At the same time, they can simultaneously cause a variety of tissue reactions, including cell stimulation, production of reactive oxygen species (ROS), inflammation, and apoptosis^48,49^. To enhance the dressings for skin wounds, GNPs have been used due to their ability in reducing the septic phase of healing through antioxidant activity^50^, promoting the migration of epithelial and mesenchymal cells in the injured skin, differentiating myofibroblasts, and quickening the angiogenesis cycle^51^. Interestingly, our data indicated that GNPs could induce more immunoregulatory effects, and improvement of mange immune factors such as IL-1, IL-6, TGF-1β, and IL-10 compared to AgNPs. Cytokines, such as IL-10 and IL-6, have been demonstrated to have a significant role in the control of typical immune responses in addition to IFN-γ^52^. The cytokine IL-10 is a crucial regulatory cytokine and mediator of anti-inflammatory processes that safeguard a host against exaggerated reactions to infections. It also plays crucial roles in a variety of other contexts, including sterile wound healing, autoimmune reaction, cancer, and homeostasis^53^. Upregulation of IL-10 is correlated to the blockage of Th1 cells and macrophage activation. IL-10 is crucial for the survival of the parasite within macrophages as well as for the control of the inflammatory response. Moreover, Hu et al.^54^ reported that the expression of toll-like receptor2 was correlated with the levels of IL-10. IFN-γ is crucial for macrophage-mediated anti-parasitic clearance, which helps to clear the parasite and end the infection^55^. Moreover, the primary mediator of iNOS transcription and nitric oxide (NO) generation following infection is IFN-γ which resulted in cell death^56^. This was confirmed also by immunohistochemistry as the less positive reaction of iNOS in histological sections of skin in both nanoparticle-treated groups.

Our results showed up-regulation of IL-10 in both Ag and GNPs treated groups. AgNPs could elevate the levels of IL-10 in rabbit serum which supports the hypothesis that AgNPs increase Th2 cells, whereas another report showed that AgNPs increase the anti-inflammatory cytokine IL-10^57^. Gold nanoparticles boosted the production of IFN-γ and L-10^58^. Administration of GNPs downregulated pro-inflammatory cytokines and chemokines, and eventually prevented the main pathological changes. Kurtjak et al.^59^ showed that AgNPs displayed excellent bactericidal activity against *Pseudomonas aeruginosa* and reduced the in vitro cytotoxicity. AgNPs could improve the immune response of mice against infection by elevating the serum levels of IL-10 and INF-γ^60^. Moreover, AgNPs increased collagen expression from dermal fibroblasts which promote the healing of skin lesions^61^. Our findings showed up-regulation of the TGF-1 along with decreasing the Cox-2 immunoexpression in all NPs treated groups. It is noteworthy to mention that TGF-1 is the key element in controlling fibroblast activation and proliferation since it up-regulates Cox-2 expression in fibroblasts^62^. Thus, Cox-2 expression is downregulated when TGF-1 is silenced^63^. Cox-2 immunoreactivity in nanoparticles treated groups was lower than the untreated group in the skin sections. Both iNOS and Cox-2 were applied as immunohistochemistry markers for inflammation^64^, therefore we observed higher immunoreactivity in the nanoparticles-treated group. Different nanoparticles have been reported to induce inflammatory reactions in various organs associated with positive immunoreactivity against Cox-2 and iNOS^65–67^. TNF-α was also expressing stronger immunoreactivity in skin tissue sections of the nanoparticles treated groups, which was also reported in previous studies on silver nanoparticles^60^. TNF-α as a proinflammatory marker has a role in the upregulation of iNOS and Cox-2^68^.

Regarding nanoparticle residues analysis, muscles from both treated groups did not show any silver or gold residues, which renders the meat from the treated rabbits fit for human consumption. The route and dosage of nanoparticles play a crucial role in nanoparticle aggregation and accumulation within tissue and organs. In a previous study, a high dosage of injected silver nanoparticles leaves residues in rabbits’ meat^19^. Moreover, silver residues were found in the muscles of broiler chickens after oral administration of silver nanoparticles^69^. In our study, silver nanoparticle residues were found only in the liver but not in the muscles. We did not detect any gold residues in the muscles and livers of the treated groups. On the contrary, it was reported that the liver and spleen accumulate the highest proportion of gold after injections of rabbits with gold nanoparticles^20^.

This study has a variety of limitations that could be incorporated into further experimental studies to confirm the safety of both silver and gold nanoparticles on rabbits. First, our study was performed on rabbits that were naturally infected with generalized sarcoptic mange. So, we applied either AgNPs or GNPs topically on the infected skin area and also subcutaneously injected to elevate the immunity of rabbits and improve their health condition. It has yet to be examined the potential effect of both nanoparticles if applied either topically or via injections alone on rabbits infected with mange less than the infections used in this study to minimize the nanoparticles exposure as possible. Second, our study was conducted on naturally infected rabbits and so we didn’t incorporate control uninfected groups either with or without NPs treatments to minimize the number of rabbits, further experimental studies were required to investigate the effect of both AgNPs and GNPs on normal rabbits if applied at the same dose and routs. Last, we focused on measuring NPs residue in the most edible parts including meat and liver (most sites of both NPs aggregation), further studies are required to measure NPs residue in other organs.

## Conclusion

In conclusion, both AgNPs and GNPs showed effective acaricidal activity against rabbit mange in vivo. Moreover, both NPs treatments resulted in the alleviation of mange-induced histopathological lesions in the skin which was confirmed with immunohistochemistry as well. Rabbit muscles were free from residues of nanoparticles which render them fit for human consumption. However, the livers of the AgNPs-treated group contained traces of silver. GNPs are recommended for the treatment of rabbit mange due to their acaricidal activity and their role in promoting skin healing without leaving residues in the animal meat and liver.

## Data Availability

All data generated or analyzed during this study are either included in this published article and its supplementary materials file (or available from the corresponding author upon reasonable request). The nucleotide sequences of partial sequence of 16S rRNA *Sarcoptes scabiei* gene obtained in this study was deposited in the National Center for Biotechnology Information (NCBI) repository (https://www.ncbi.nlm.nih.gov/nuccore/ON241779.1/) under accession number ON241779.
